# Do political factors matter in explaining under- and overweight outcomes in developing countries?

**DOI:** 10.1016/j.socec.2013.06.002

**Published:** 2013-10

**Authors:** Elena Fumagalli, Emmanouil Mentzakis, Marc Suhrcke

**Affiliations:** a Institute of Health Economics and Management (IEMS) and Department of Economics (DEEP), University of Lausanne, Switzerland; b Economics Division, School of Social Sciences, University of Southampton, Southampton, UK; c Health Economics Group, Faculty of Medicine and Health Sciences, University of East Anglia, Norwich Research Park, Norwich, UK; d UKCRC Centre for Diet and Physical Activity, Institute for Public Health, Cambridge, UK

**Keywords:** BMI, Obesity, Developing countries, Generalized ordered response models

## Abstract

We construct a rich dataset covering 47 developing countries over the years 1990–2007, combining several micro and macro level data sources to explore the link between political factors and body mass index (BMI). We implement a heteroskedastic generalized ordered logit model allowing for different covariate effects across the BMI distribution and accounting for the unequal BMI dispersion by geographical area. We find that systems with democratic qualities are more likely to reduce under-weight, but increase overweight/obesity, whereas effective political competition does entail double-benefits in the form of reducing both under-weight and obesity. Our results are robust to the introduction of country fixed effects.

## Highlights

• We study the role played by political factors in shaping the BMI distribution.
• We allow for differing covariate effects across the BMI distribution.
• We allow for heteroskedasticity across macro regions.
• Democratic systems reduce under-weight, but increase overweight/obesity.
• Effective political competition reduces both under-weight and obesity.

## 1 Introduction

The bulk of the public health and economic research on developing countries traditionally focused on communicable, maternal, perinatal, and nutritional conditions (Behrman et al., 2009), with under-nutrition generally considered to be the most important nutritional challenge in low- and middle-income countries. However, it has recently been acknowledged that the “obesity epidemic” has increasingly spread in developing countries (Monteiro et al., 2004; Popkin, 2001, 2008), while under-nutrition still continues to prevail, producing a “double burden of malnutrition” (Lukito and Wahlqvist, 2006; Prentice, 2006). Inspired by Besley and Kudamatsu's (2006) work on political factors and mortality rates, we investigate the effect of political factors on body mass index (BMI),^1^ a measure of the human body shape often used as a proxy for adiposity. To our knowledge, this is the first paper that examines the effect of political factors on the BMI distribution using a rich micro-level dataset, controlling for a range of confounding factors and covering a large number of developing countries.

We construct a dataset of 47 developing countries from 1990 to 2007, containing individual socio-economic characteristics collected in the Demographic and Health Survey (DHS), as well as demographic and country specific information from different data sources. We use a categorical version of the BMI variable (i.e. underweight, normal weight, overweight or obese), and analyze the entire BMI distribution not restricting our analysis to obese (e.g. Maennig et al., 2008) or underweight (e.g. Milman et al., 2005) individuals. By studying the top, middle and bottom parts of the distribution we are uniquely able to assess whether political factors affect over- and underweight in different ways.

**Fig. 1:**
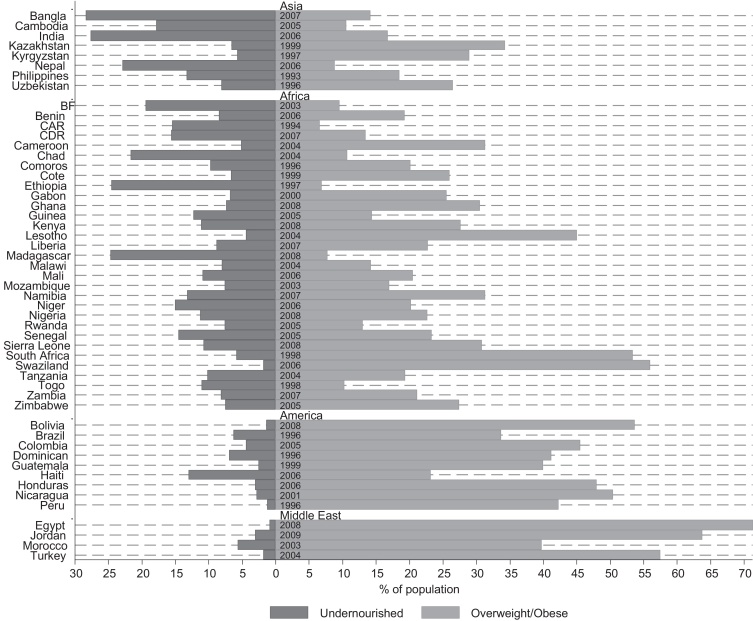
The prevalence of under- and overweight/obesity in 47 countries of the DHS (women, aged 18–49).

Since several of the countries in our sample are facing a double burden of both over- and under-weight (especially in Asia and Africa – see Fig. 1), we exploit an econometric specification which allows for the identification of distinct links between the various points of the BMI distribution and the covariates of interest. Further, given the use of a cross country dataset, we allow for heterogeneity by geographical areas (i.e. modifying the estimator to fit a heteroskedastic specification), as the commonly used BMI definition for obesity (i.e., BMI > 30) might not be an appropriate measure of fatness for all ethnic groups (Barba et al., 2004). We further introduce country and time fixed effects in the estimations to account for unobserved country specific factors, time invariant characteristics and macro time-specific shocks.

In short, we find that political characteristics are important in determining BMI status, even after controlling for relevant covariates as well as country fixed effects. In particular, we find that systems with democratic qualities are more likely to reduce under-weight, but increase overweight/obesity, whereas effective political competition does entail double-benefits by reducing both under-weight and obesity (increasing the chances of being normal weight). The heteroskedastic specification suggests a significant difference in the variance of BMI across geographical areas, with Asian countries showing the lowest variability.

The paper is structured as follows. In Section 2 we present the background of the research. Section 3 describes the data and Section 4 the econometric specification. Finally, Sections 5 and 6 contain the description of the results and the discussion.

## 2 Background

Ecological studies have shown that political freedom (Alvaro et al., 2004; Franco et al., 2004) and democratization (Kudamatsu, 2012) are positively associated with different measures of health, e.g. maternal and infant mortality. Similarly, Vollmer and Ziegler (2009) find a positive association between democratization and human development (measured by literacy and life expectancy), and Klomp and De Haan (2009) establish a positive relationship between democracy and a composite individual health index. By contrast, Young (1990) fails to find empirical support for Owens’ hypothesis (Owens, 1987) that populist authoritarian regimes increase physical quality of life more than “false democracies”^2^ do. Besley and Kudamatsu (2006) find that the relationship between life expectancy and democratization is not robust to the inclusion of country fixed effects, suggesting that political variables might capture some unobserved country level factors and not just the effect of democratization itself. Such concerns are reinforced in light of the fact that – with the exception of Kudamatsu (2012) – all of the above studies investigate the impact of political characteristics on health outcomes at the aggregate country level.

While the above-described body of evidence does not focus on BMI outcomes, potentially relevant insights may still be inferred for the present paper. There are multiple channels through which political characteristics might in principle affect health. First, democratic governments may be more efficient (Adam et al., 2011) and political parties with egalitarian ideologies, or proportional systems could provide a greater incentive for designing pro-poor public health policies, compared to the incentives provided in autocratic regimes (see Mulligan et al., 2004; Navarro et al., 2006; Wigley and Akkoyunlu-Wigley, 2009). Second, in democratic regimes candidates compete for voting, and hence the “quality” of the elected policy-makers and of the health (and other) policies they implement might be superior to that in authoritarian countries (Ruger, 2005; Besley and Kudamatsu, 2006). Third, non-autocratic regimes may be better placed to translate economic growth into a higher amount of calories (Blaydes and Kayser, 2011). Competitive elections encourage candidates and incumbent governments to attract voters with targeted rewards and the most effective voters to target are the poor, through pro-poor policies that result in greater amounts of food (which would reduce under-nutrition but may increase obesity).

The above theoretical mechanisms should lead us to expect that democracy aids in reducing at least under-nutrition, while the expected effect of democracy or other political factors on obesity in countries that have exceeded a minimum level of food availability is ambiguous. To the extent that obesity reflects the rational, free choice of individuals (Philipson and Posner, 2003), autocratic regimes that have an interest in their population's health (of which there may not be too many) may, at least in principle, be better placed to enforce healthier lifestyles through various regulatory measures (Toh et al., 2002).

Only Besley and Kudamatsu (2006) have explicitly analyzed the role of political characteristics in relation to under-nutrition. In the explanation of the under-nutrition phenomenon, behavioral factors are arguably much less important than for obesity, since the trade-off between the pleasure from (over-)consumption of food and maintaining a healthy weight does not apply. Hence, under-nutrition is less likely to be a condition caused by preferences, but mostly by binding (mainly economic) constraints restricting the provision of and access to food. Hence, the role of political factors could, in principle, be very relevant. There is considerable evidence on the fundamental importance of the spatial and environmental (Kandala et al., 2001; Cleah et al., 2010; Khatab, 2010; Lovadino de Lima et al., 2010) as well as the economic determinants (Heltberg, 2009) of under-nutrition, while individual characteristics and preferences appear to matter less. Investigating the determinants of child stunting, Milman et al. (2005) assessed the importance not only of individual characteristics (e.g. food insecurity, insufficient purchasing power), but also of aggregate types of determinants, with the latter ones (specifically the change in immunization rate, safe water availability, energy supply, initial female literacy rate, and degree urbanization) estimated to be the most important. It is worth noting that the significance found for the aggregate influences might, at least partially, be attributable to the lack of control for fixed effects.

While evidence on the impact of political settings on obesity in developing countries is scarce, important work on the more “standard” determinants informs our choice of covariates. Dinsa et al. (2012) find a positive association between socioeconomic status (SES) and obesity for both men and women in low income countries, while for middle income countries the association becomes largely mixed for men and mainly negative for women. Further empirical findings from single country studies indicate that childhood deprivation and higher SES significantly increase the likelihood of being obese in urban Africa and Malaysia (Case and Menendez, 2009; Tan et al., 2012). Looking at under- and over-weight Corsi et al. (2011) for Bangladesh argue that household and neighborhood wealth is negatively (positively) related to being underweight (overweight), with residence in rural neighborhoods being significantly associated with decreased levels of overweight.

## 3 Data and variables considered

This study employs a rich set of data (see Tables 1 and 2 for aggregate descriptive statistics), combining both micro and macro level information, and covering 47 developing countries for 17 (1991–2007) years, with each country appearing between one and five times (Table A1). The individual microdata come from different waves of the Demographic and Health Surveys (DHS), which are nationally representative repeated cross-sectional surveys covering different aspects of health and demographics. The DHS focuses largely on women in fertile age, usually 15–45 years old. In almost all countries height and weight are measured by the interviewer, hence avoiding reporting bias and allowing for objective BMI measures to be derived. We create a global dataset by arranging all the countries and years in which such information was included.

Further, the dataset is enriched by country-level information from four additional sources: (a) country level political characteristics are taken from the Polity IV Project database, which covers all major independent states world-wide,^3^ (b) economic indicators are obtained from the World Bank's World Development Indicators (WDI), (c) economic and institutional characteristics come from the Cross-National Time-Series Data Archive (CNTS),^4^ which provides annual data for a range of countries from 1815 to the present, and (d) proxies of nutritional habits (e.g. total average fat intake) are taken from FAOSTAT, which provides time-series and cross sectional data relating to food and agriculture for some 200 countries from 1960.^5^

Using the WHO BMI classification, individual BMI is transformed into a categorical measure taking four values: underweight (<18.5), normal weight (18.5–24.99), overweight (25.00–29.99) and obese (>30). Table 1 provides average proportions calculated across all countries. To correct for racial disparity in BMI classification, we allow for heteroskedasticity by macro-region (America, Africa, Asia, Middle East).

Out of our set of explanatory variables, the definitions of the political variables, which are the primary interest of our study, warrant further discussion. Since the literature has focused both on the effect of the type of government and on the way political competition takes place, we chose two variables to capture the political characteristics of a country. Following Persson and Tabellini (2004) and Besley and Kudamatsu (2006), democracy is defined as a dichotomous variable equal to one (=democratic), if the POLITY2 score is positive, and zero otherwise.^6^ POLITY2 is a slightly modified revision of the POLITY score, which is a “convenient avenue for examining general regime effects in analyses”, and it measures the “concomitant qualities of democratic and autocratic authority in governing institutions” (Marshall et al., 2010, p. 17). In addition, following Besley and Kudamatsu (2006), we argue that one of the major factors influencing health (and thus BMI in our case) is the quality of the political competition, which in principle should ensure that the most competent policy makers will be selected. Therefore, we include in our analysis the quality of the executive competition. The indicator is based on the EXREC variable of the Polity IV database, which takes eight values from “Succession by birthright” to “Formal competition among publicly supported candidates”. We dichotomize our measure of executive competition, and give the value of 1 if EXREC is 6 (“Ascriptive and elective rulers coexist”) or higher (see also Besley and Kudamatsu, 2006), thus capturing the cases in which some electoral competition does exist.

To control for confounding and to isolate the effect of political characteristics on BMI, a number of covariates are introduced in the estimation (see Table 2), both at the individual (i.e. education, age and its square, living in an urban area, and working status) and at the aggregate level. To distinguish the effect of political development from the one of economic development, we also include measures of the economic wealth of a country (i.e. the level and growth rate of per capita GDP), a measure of how “globalized” the economy is (i.e. imports of goods and services as % of GDP), the quality of its services (e.g. number of telephones and physicians per 1000 people) and the average quality of the diet (e.g. proportion of fat in diet).

Furthermore, changes in individual BMI are likely to respond to country level changes of the past – a feature we account for by introducing three-year moving averages (including current year and with equal weight across all three years) for all country level covariates. Therefore country level binary covariates are transformed into the proportion of, for example, democratic years over the three years (i.e. the current one plus the past two).

Finally, we restrict our sample to individuals of 18 years old and over, to avoid the idiosyncratic patterns exhibited by students and adolescents. Table 2 provides the descriptive statistics, in terms of averages across women in all countries and all years, for the variables included in the analysis.

## 4 Econometric model and estimation

Using the ordinal nature of the BMI variable and assuming BMI to be a linear function of the covariates that is logistically distributed, the ordered response logit models can be written as

(1) $$\mathrm{Pr}(BMI_{i}\leq j)=\Lambda (X_{i}\beta )=\frac{\exp(\alpha_{j}-X_{i}\beta )}{1+\exp(\alpha_{j}-X_{i}\beta )}$$

where *j* = 1, …, *J* is the number of BMI categories (in our case 4:1 = Underweight, 2 = Normal, 3 = Overweight, 4 = Obese) and *i* = *1*,…,*n* is the individuals in the sample. The vector *X*_*i*_ indicates the covariates used in the estimation, while *a*_*j*_ are the cut-off points to be estimated along with the rest of the parameters.

Such specification constrains all parameters (apart from the alphas) to be equal across all BMI categories. This assumption is referred to as single crossing property and requires that as one moves from the probability of the smallest outcome to the probability of the largest outcome, the marginal probability effects are allowed to change their sign (effect) once^7^ (Boes and Winkelmann, 2006). Borrowing from Long and Freese (2005), the ordered response model is equivalent to *j* − 1 binary regressions, assuming that the slope coefficient for each covariate is constant across regressions (i.e. parallel lines assumption).

Relaxation of this assumption can be carried out by making the cut-off points linear functions of the covariates, and as such introduce heterogeneity in the coefficients (Terza, 1985). Substituting *a*_*ij*_ = *k*_*j*_ + *X*_*i*_*γ*_*j*_ in Eq. (1) gives the generalized model,

(2) $$\mathrm{Pr}(BMI_{i}=j|X_{i})=\Lambda (\kappa_{j}-X_{i}\beta_{j})-\Lambda (\kappa_{j-1}-X_{i}\beta_{j-1})$$

where the estimated coefficients are *β*_*j*_ = *γ*_*j*_ − *β*.

A further common assumption of the model is its assumed homoskedasticity, which we relax by parameterizing the variance (*σ*) and making it a function of observable characteristics. As past literature argues (Barba et al., 2004), the commonly used BMI definitions and BMI cut-offs are not appropriate measures of adiposity for all ethnic groups and geographical areas, in particular for Asian countries. To accommodate this, we allow for heteroskedasticity according to region (i.e. Asia, Africa, America and Middle East) of the form *σ*_*i*_ = exp(*z*_1_ · Asia + *z*_2_America + *z*_3_MiddleEast), where Africa is the reference category with *σ*_*i*_ = exp(0) = 1 and the rest of the scales (i.e. *z*) are estimated.

In the estimations we control for country and time fixed effects, while we also add as covariates the country mean of all individual characteristics to account for between country heterogeneity (Mundlak, 1978). Following the estimation, marginal effects^8^ are computed in order to be able to compare the magnitude of the coefficients.

## 5 Results

### 5.1 Sample descriptive statistics

Table 2 presents sample descriptive statistics. About 60% of the individuals included in our sample have a normal BMI, while 13, 18 and 9% are underweight, overweight or obese, respectively. The mean age is 31 years, with 29% of the respondents reporting complete primary/incomplete secondary education and 16% completed secondary or higher education, while about 50% of them work and 43% reside in an urban area.

In terms of country characteristics, an average of 2 years (of the last three) have been spent under democratic regimes, and about the same length of time have been spent in regimes with a competitive executive nominating process. In term of economic characteristics, the reported mean three-year moving average GDP per capita is $2738, with imports of goods and services covering 34% of the GDP. The average proportion of fat in the diet (over three years) is at about 18%.

### 5.2 Estimation results

Table 3 gives the signs and magnitudes of the marginal effects of the various covariates across the four categories of the BMI distribution. As for the two political indicators – our independent variables of primary interest – the effects vary considerably across the BMI distribution. It appears that individuals in democratic countries (compared to non-democratic ones) are more likely to be overweight and obese, i.e. by 2.5 p.p. and 1.5 p.p., respectively, but also less likely to be underweight by about 1.5 p.p. By contrast, the quality of political competition appears to be beneficial for both the reduction of underweight (by 1.6 p.p.) and of obesity (by 1.5 p.p.).

The other covariates also offer interesting insights: As expected, individuals in countries with higher per capita GDP face a significantly reduced risk of being underweight and an increased probability of being overweight (although the variable is not statistically significant for obesity) with a percentage increase in GDP increasing the odds of being overweight by 0.2 p.p., while reducing underweight probability by approximately 0.06 p.p. By contrast, a percentage increase in annual per capita GDP growth reduces the probability of being overweight or obese by 0.2 and 0.25, respectively, while it increases the probability for individual normal BMI by 0.4 p.p. and appears to have no significant impact on underweight. Finally, looking at an indicator of the level of globalization and openness of the economy, devoting an extra 10% of GDP toward imports of goods and services, will decrease the probability for normal BMI by 1.2 p.p.

The number of physicians, that we include as a proxy of the quality of the health system and as a proxy of the level of investment in health, to capture the fact that democracies tend to spend more on healthcare (Besley and Kudamatsu, 2006), has no significant effect on obesity but does increase overweight by 4 p.p. for every extra physician per 1000 people, while it decreases the probability for underweight and normal weight by a similar combined percentage (i.e. 2.1 p.p. and 1.5 p.p., respectively). On the other hand, the number of telephones per 1000 people, introduced as a proxy of technological development, has statistically significant effects: it reduces the probability of being underweight as well as the probability of being obese while increasing the probability of being normal weight. However, the magnitude of the effect is very small (e.g. there is an about 0.15 p.p. and 0.01 p.p. decrease in underweight and obesity probability, respectively, for every 10 extra phones per 1000 people).

All educational levels (compared to no education) tend to increase the likelihood of being overweight or obese and decrease the likelihood of being underweight and normal weight. Education seems to have a linear effect, with the absolute effect magnitude increasing in size as we go from lower to higher levels of education. As expected from the literature on obesity (e.g. Reither et al., 2009) age displays a bell-shaped effect, with the probability of overweight and obesity initially increasing with age and then decreasing once a certain age threshold is reached. The opposite holds for normal- and under-weight. Moreover, living in an urban area also tends to reduce (increase) the probability of being underweight (normal and overweight or obese) by similar amounts. Working women have a reduced probability of belonging to all categories apart from normal BMI, which increases by about 2 p.p.

The nutritional pattern, proxied by the (national average) percentage of fat in the diet, is significantly associated with the individual probability of being normal or overweight/obese, but not with underweight: a 10% increase in the percentage of fat in the diet increases the likelihood of normal BMI by 7.6 p.p., while it reduces the probability of being overweight and obese by almost 6 p.p. and 2 p.p., respectively.

Finally, the parameterization of the variance was significant, indicating non-negligible variability in the estimates by geographical location and underlining the importance of our correction for heteroskedasticity. As expected, compared to Africa, Asian populations seem to have significantly less variability in the BMI outcomes, while the variability is higher than the reference group, when considering individuals living in American and Middle Eastern countries.

## 6 Discussion

Using a global perspective and employing a large dataset covering 47 developing countries, this paper is the first to use such a rich and extensive dataset to examine the impact of political factors on nutritional status (proxied by BMI) at the individual level. With obesity and malnutrition rapidly becoming global problems affecting the developing world, this paper adds to the general literature on the role of political characteristics in determining health. Owing to our flexible econometric specification, the links between the entire distribution of BMI (i.e. underweight to obese) and the quality of the government could be examined.

Even after controlling for a large set of individual and country specific covariates and country fixed effects, our analysis shows that political factors at the country level do explain a significant part of the variation in the BMI status. The influence of the “quality” of the political system (i.e. our executive competition variable) in a country confirms the idea that it may not only be the type of government that determines health, but also the way political competition works. Our findings are in line with the broadly related literature suggesting that democratic countries seem to be able to increase the amount of calories consumed, as argued by Blaydes and Kayser (2011), thus shifting the whole BMI distribution to the right. The effect of such a shift is the reduction of the burden of under-nutrition and the increase of the percentage of people who are both overweight and obese. Such decrease in under-weight is also in line with the Besley and Kudamatsu (2006) theory and with the empirical results of Smith and Haddad (1999) who find that a unitary increase in democracy (on a 1–7 scale) leads to a drop in the prevalence of child under-nutrition by about 0.8–1.3 p.p. in developing countries. At the same time, however, our results do qualify the finding from previous literature of the alleged general health improving effects of democracy (Besley and Kudamatsu, 2006). Klomp and De Haan (2009) showed, for instance, that a 1% increase in democracy improved the health of individuals (measured as generic health) by 0.13%. According to our results, however, democracy may be good for many health aspects, but it may also have adverse effect in the form promoting obesity. It might be the case that democratic low- and middle-income countries, as defined in our variable, are more similar to the western democracies, where a nutritional culture based on the consumption of energy dense food and the diffusion of fast foods have become very popular in the recent years.

By contrast, our results regarding the variable measuring the efficiency and freedom in the choice of politicians and policy makers support the idea that competition between politicians ultimately promotes the implementation of better (health) policies, at least as far as the health proxies considered here are concerned. We specifically find that improvements in the quality of the political competition are beneficial in curtailing the tails of the BMI distribution. Countries whose electoral mechanisms work effectively are characterized by a lower prevalence of both malnutrition and obesity. Our estimated drop in obesity by 1.5 percentage points is close in magnitude to the effect obtained by Yoon and Brown (2011) who looked at social capital and obesity, finding a twofold increase in the ratio of employees in voluntary organizations to the total population to reduce the likelihood of being obese by between 1.4 and 3.3 percentage points. It is worth noting that our measures of political performance, contrary to Besley and Kudamatsu's (2006) study, are not only robust to the introduction of other variables at the country level (e.g. GDP, average level of education), but also to the introduction of country fixed effects.

One basic motivation for our analysis was to ideally identify factors that either could help tackle both under- and overweight at the same time, or at least one of them without harming the other. If this was the case, and if those factors were amenable to policy intervention, then this would lead to fairly straightforward policy implications, even in countries facing a double burden of under- and overweight. Unfortunately, our results suggest that this ideal scenario is the exception rather than the rule. For a number of factors we find a beneficial effect on one type of malnutrition coupled with an adverse effect on the other. This holds for our indicator of democracy and for other controls such as the level of education, urbanization, GDP per capita and the number of physicians per capita.

The estimated effect for education in terms of decreasing the probability of being underweight is in line with previous results (Lovadino de Lima et al., 2010), while the exposure to greater risk of overweight and obesity in developing countries is also in line with the public health literature (Monteiro et al., 2004) that found that at least for the low income countries it is those with higher education that bear the greatest burden of obesity. In our bigger sample, we do not, however, confirm the non-linearities detected in their paper.

The increased probability of being overweight and obese for people living in urban areas and in more open economies confirms the hypothesis that people more exposed to western culture are more likely to be obese. This is akin to the idea that globalization tends to stimulate an obesogenic environment in developing countries (Hawkes, 2006), fueled by the increase in the availability of energy dense food (see e.g. Currie et al., 2010). Moreover, our finding that the percentage of fat in total calorie intake decreases overweight and obesity and increases underweight but leaves normal BMIs unaffected supports Popkin's (2008) idea that the main force that drives obesity is not the diet composition, but rather the positive balance between calorie intake and consumption.

Looking at the number of physicians, used as a proxy for health care quality, we find a reduction in the likelihood of underweight and an increase for overweight. In some cultures being overweight does not carry negative connotations, but is instead seen as a sign of being wealthy that may not require immediate medical intervention (Case and Menendez, 2009), in contrast to underweight. However, caution is suggested in interpreting this particular finding, since health care quality is a multi-dimensional characteristic of the health system that may not be easily captured by a single indicator (Murray and Evans, 2003).

Interestingly, the rate of economic growth, controlling for the level of per capita GDP, and labor force participation improve the entire BMI distribution, i.e. shortening the tails. This is in some contrast to Ruhm (2000) who showed that for high income countries recessions (booms) are associated with improvements (deteriorations) in health. The under-weight-reducing effect (though insignificant in our estimates) is more in line with existing evidence, as, for instance, Heltberg (2009), who concludes that economic growth decreases stunting, but at a slow pace. The effect observed for employment might be an indication that underweight, overweight and obese individuals face discrimination (or face actual health problems due to their weight) in the labor market and hence reduce their labor supply (Atella et al., 2008 and DeBeaumont, 2009), which is also supported by the model of Dasgupta and Ray (1986, 1987), according to which the malnourished are unemployed, because they lack physical energy but they need a high wage to “buy” a sufficient quantity of calories.

A number of limitations for our analysis need to be acknowledged. First of all, even if robust, our results do not necessarily imply a causal link between political factors and BMI. And even if causal inference was allowed, it is important to bear in mind that the changes in the political system that would be required to achieve a notable reduction in malnutrition would be quite substantial (i.e. a shift from a non-democratic to a democratic system, or from a system with poor political competition to one with a high level). Nevertheless, our findings confirm, strengthen and add some nuance to the correlation found by Besley and Kudamatsu (2006).

Further, due to data constraints, we only employ an aggregate measure of economic status (GDP per capita), without being able to include an individual income measure in the analysis. In fact, in developing countries it is very difficult to correctly identify rich and poor individuals given that wage income fails to capture the true material wealth of a household, as many households rely on self production. A more widely used and likely more meaningful measure of households’ economic wealth is an asset-based wealth index (Filmer and Pritchett, 2001), constructed using a factor analysis based on the ownership of a set of assets. We decided not to include this indicator for a number of reasons: first, it measures relative wealth, given that by construction it has mean zero and standard deviation equal to one. Hence, it does not capture increased purchasing power; second, the number and characteristics of the assets included in the DHS questionnaire tends to vary between waves, thereby severely compromising comparability between surveys. Given the high correlation between individual educational level and income in the developing countries it was decided that education could be left to act as a proxy for socio-economic status of the individuals.

Despite the lack of available data to address such caveats, the global perspective adopted in this paper and the rich econometric specification allow for the study of the effect of political factors on the full range of the BMI distribution, while accounting for a wide range of influencing factors, country fixed effects and potential regional heterogeneity of BMI. It constitutes the first study of its kind and prepares the ground for more in-depth exploration of the issue.

## Figures and Tables

**Table A1:** Number of observations by Country and Year.

Country/Year	1991	1992	1993	1994	1995	1996	1997	1998	1999	2000	2001	2002	2003	2004	2005	2006	2007	Total
BF	0	0	3389	0	0	0	0	0	3290	0	0	0	9332	0	0	0	0	16,011
Bangla	0	0	0	0	0	0	3765	0	0	4343	0	0	0	9819	0	0	9687	27,614
Benin	0	0	0	0	0	2262	0	0	0	0	4745	0	0	0	0	13,064	0	20,071
Bolivia	0	0	0	2283	0	0	0	4117	0	0	0	0	13,919	0	0	0	0	20,319
Brazil	0	0	0	0	0	3071	0	0	0	0	0	0	0	0	0	0	0	3071
CAR	0	0	0	1930	0	0	0	0	0	0	0	0	0	0	0	0	0	1930
CDR	0	0	0	0	0	0	0	0	0	0	0	0	0	0	0	0	3642	3642
Cambodia	0	0	0	0	0	0	0	0	0	5903	0	0	0	0	6702	0	0	12,605
Cameroon	0	0	0	0	0	0	0	1545	0	0	0	0	0	3902	0	0	0	5447
Chad	0	0	0	0	0	0	3554	0	0	0	0	0	0	2828	0	0	0	6382
Colombia	0	0	0	0	3226	0	0	0	0	3152	0	0	0	0	29,809	0	0	36,187
Cote	0	0	0	2978	0	0	0	0	2274	0	0	0	0	0	0	0	0	5252
Dominican	2095	0	0	0	0	6396	0	0	0	0	0	0	0	0	0	0	0	8491
Egypt	0	4808	0	0	6695	0	0	0	0	13,860	0	0	8133	0	17,179	0	0	50,675
Ethiopia	0	0	0	0	0	0	5233	0	0	0	0	0	0	0	0	0	0	5233
Gabon	0	0	0	0	0	0	0	0	0	2301	0	0	0	0	0	0	0	2301
Ghana	0	0	1752	0	0	0	0	2033	0	0	0	0	4288	0	0	0	0	8073
Guatemala	0	0	0	0	4833	0	0	0	2304	0	0	0	0	0	0	0	0	7137
Guinea	0	0	0	0	0	0	0	0	3188	0	0	0	0	0	3068	0	0	6256
Haiti	0	0	0	1837	0	0	0	0	0	7774	0	0	0	0	0	4104	0	13,715
Honduras	0	0	0	0	0	0	0	0	0	0	0	0	0	0	0	15,223	0	15,223
India	0	0	0	0	0	0	0	0	0	0	0	0	0	0	0	99,777	0	99,777
Jordan	0	0	0	0	0	0	3062	0	0	0	0	0	0	0	0	0	4489	7551
Kazakhstan	0	0	0	0	3119	0	0	0	1990	0	0	0	0	0	0	0	0	5109
Kenya	0	0	3284	0	0	0	0	3215	0	0	0	0	6198	0	0	0	0	12,697
Kyrgyzstan	0	0	0	0	0	0	3115	0	0	0	0	0	0	0	0	0	0	3115
Lesotho	0	0	0	0	0	0	0	0	0	0	0	0	0	2686	0	0	0	2686
Liberia	0	0	0	0	0	0	0	0	0	0	0	0	0	0	0	0	5362	5362
Madagascar	0	0	0	0	0	0	2504	0	0	0	0	0	0	6344	0	0	0	8848
Malawi	0	2263	0	0	0	0	0	0	0	9943	0	0	0	8530	0	0	0	20,736
Mali	0	0	0	0	0	4086	0	0	0	0	9196	0	0	0	0	10,594	0	23,876
Morocco	0	2851	0	0	0	0	0	0	0	0	0	0	13,862	0	0	0	0	16,713
Mozambique	0	0	0	0	0	0	3095	0	0	0	0	0	9209	0	0	0	0	12,304
Namibia	0	2195	0	0	0	0	0	0	0	0	0	0	0	0	0	0	7588	9783
Nepal	0	0	0	0	3323	0	0	0	0	7658	0	0	0	0	0	8669	0	19,650
Nicaragua	0	0	0	0	0	0	0	10,237	0	0	10,138	0	0	0	0	0	0	20,375
Niger	0	3191	0	0	0	0	0	3225	0	0	0	0	0	0	0	0	0	6416
Nigeria	0	0	0	0	0	0	0	0	1926	0	0	0	5614	0	0	0	0	7540
Peru	5106	0	0	0	0	10,605	0	0	0	0	0	0	0	0	0	0	0	15,711
Rwanda	0	0	0	0	0	0	0	0	0	7558	0	0	0	0	4367	0	0	11,925
Senegal	0	0	2842	0	0	0	0	0	0	0	0	0	0	0	3413	0	0	6255
Swaziland	0	0	0	0	0	0	0	0	0	0	0	0	0	0	0	3822	0	3822
Tanzania	4397	0	0	0	0	3734	0	0	0	0	0	0	0	7835	0	0	0	15,966
Togo	0	0	0	0	0	0	0	3257	0	0	0	0	0	0	0	0	0	3257
Turkey	0	0	2393	0	0	0	0	2302	0	0	0	0	0	3010	0	0	0	7705
Uzbekistan	0	0	0	0	0	3488	0	0	0	0	0	0	0	0	0	0	0	3488
Zambia	0	3168	0	0	0	3783	0	0	0	0	0	5781	0	0	0	0	5344	18,076
Total	11,598	18,476	13,660	9028	21,196	37,425	24,328	29,931	14,972	62,492	24,079	5781	70,555	44,954	64,538	155,253	36,112	644,378

**Table 1:** Aggregate percentages for non-normal BMI by geographical region.

	Underweight BMI	Overweight/Obese BMI
Africa	11.41	17.07
America	4.13	42.11
Middle East	2.01	51.52
Asia	26.40	15.12

**Table 2:** Sample descriptive statistics.

	Mean/proportion^a^	Std. Dev.
**Country characteristics**		
*Political situation*		
Democracy^1^	0.657	0.460
Executive Competition^1^	0.628	.462
*Development*		
# of physician per 1000 people^2^	0.644	0.767
#of telephones per 1000 people^2^	46.2	58.3
Financial position		
GDP per capita, PPP^3^	2738.5	2258.9
Annual GDP growth (%)^3^	0.026	0.034
Imports of goods and services as % of GDP^3^	34.3	18.3
Nutritional habits		
Proportion of fat in diet^4^	0.182	0.045

**Individual characteristics**		
*BMI*		
Underweight	0.126	
Normal	0.596	
Overweight	0.186	
Obese	0.093	
*Education*		
Incomplete primary	0.204	
Complete primary/incomplete secondary	0.285	
Complete secondary or higher	0.159	
Age	30.8	8.95
Living in urban area	0.426	
Working	0.501	

a The mean for binary indicators implies sample proportions. *Data sources*: ^1^ Polity IV, ^2^ CNTS, ^3^ WDI, ^4^ FAOSTAT.

**Table 3:** Marginal effects (computed at the mean) for the generalized heteroskedastic ordered logit estimation.

	Underweight	Normal	Overweight	Obese
**Country characteristics**				
*Political situation*				
Democracy	−0.0147***	−0.0260**	0.0251***	0.0155***
	(0.00460)	(0.0110)	(0.00968)	(0.00494)
Executive Competition	−0.0161***	0.0285**	0.00277	−0.0152***
	(0.00561)	(0.0118)	(0.0101)	(0.00486)
*Development*				
# of telephones per 1000 people	−0.000153**	0.000260**	−9.36e−06	−9.77e−05***
	(6.71e−05)	(0.000104)	(8.09e−05)	(3.32e−05)
# of physician per 1000 people	−0.0206***	−0.0150*	0.0383***	−0.00262
	(0.00648)	(0.00908)	(0.00671)	(0.00280)
*Financial position*				
Country log(GDP)	−0.0578***	−0.148***	0.195***	0.0103
	(0.00734)	(0.0190)	(0.0172)	(0.00865)
Annual GDP growth (%)	0.00601	0.439***	−0.200***	−0.245***
	(0.0274)	(0.0646)	(0.0561)	(0.0260)
Imports of goods and services as % of GDP	0.000188*	−0.00115***	0.000633**	0.000327***
	(0.000101)	(0.000276)	(0.000247)	(0.000125)
*Nutritional habits*				
Proportion of fat in diet	0.0401	0.751***	−0.599***	−0.192***
	(0.0641)	(0.159)	(0.141)	(0.0704)

**Individual characteristics**				
*Education*				
Incomplete primary	−0.0207***	−0.0574***	0.0591***	0.0190***
	(0.000754)	(0.00181)	(0.00160)	(0.000674)
Complete primary/incomplete secondary	−0.0312***	−0.0974***	0.0969***	0.0317***
	(0.000612)	(0.00176)	(0.00155)	(0.000691)
Complete secondary or higher	−0.0394***	−0.0851***	0.102***	0.0228***
	(0.000738)	(0.00243)	(0.00209)	(0.000785)
Age	−0.00551***	−0.0346***	0.0273***	0.0128***
	(0.000199)	(0.000587)	(0.000522)	(0.000216)
Age square	6.55e−05***	0.000355***	−0.000287***	−0.000134***
	(3.08e−06)	(8.65e−06)	(7.74e−06)	(3.21e−06)
Living in urban area	−0.0209***	−0.115***	0.0973***	0.0384***
	(0.000468)	(0.00151)	(0.00129)	(0.000605)
Working	−0.00197***	0.0206***	−0.0114***	−0.00723***
	(0.000481)	(0.00129)	(0.00117)	(0.000495)

Observations			644,378	

Standard errors computed by the Delta method in parentheses.

* *p* < 0.1.

** *p* < 0.05.

*** *p* < 0.01.

## Appendix A

See Table A1.

## Footnotes

1 BMI is defined as $\frac{\text{weight}(\text{in}\;\text{kg}).}{\text{height}{\left(\text{in}\;\text{metres}\right)}^{2}}$.

2 According to Owens, in false democracies political rights are ensured, while economic and social ones are not (Owens, 1987).

3 Produced by the Center for Systemic Peace. See http://www.systemicpeace.org/polity/polity4.htm.

4 Distributed by Databanks International.

5 See http://faostat.fao.org.

6 The adjustments made to the variable by Besley and Kudamatsu (2006) are not necessary for our purposes as this study covers a different time-span.

7 For example, if a variable had positive effect on the probability of underweight, a negative on normal and positive again on overweight and decrease, such models would be unable to detect it.

8 For continuous variable the marginal effect is *ME* = *f*(*κ*_*j*−1_ − *X*_*i*_*β*_*j*−1_)*β*_*j*−1_ − *f*(*κ*_*j*_ − *X*_*i*_*β*_*j*_)*β*_*j*_, while for discrete ones it is $ME=\mathrm{Pr}(BMI_{i}=j|{\bar{x}}_{i},w_{k}=1)-\mathrm{Pr}(BMI_{i}=j|{\bar{x}}_{i},w_{k}=0)$.
